# All together now: Simultaneous feature integration and feature retrieval in action control

**DOI:** 10.3758/s13423-021-01999-6

**Published:** 2021-10-28

**Authors:** Birte Moeller, Christian Frings

**Affiliations:** Cognitive Psychology, University of Trier, D-54286 Trier, Germany

**Keywords:** Action control, Stimulus-response binding, Response-response binding, Simultaneous binding and retrieval

## Abstract

Accounts of human action control assume *integration* of stimulus and response features at response execution and, upon repetition of some of those features, *retrieval* of other previously integrated features. Even though both processes contribute sequentially to observed binding effects in studies using a sequential prime-probe design, integration and retrieval processes theoretically affect human action simultaneously. That is, every action that we execute leads to bindings between features of stimuli and responses, while at the same time these features also trigger retrieval of other previously integrated features. Nevertheless, the paradigms used to measure binding effects in action control can only testify for integration of stimulus and response features at the first (R1, n-1, or prime) and retrieval of the past event via feature repetition at the second (R2, n, or probe) response. Here we combined two paradigms used in the action control literature to show that integration and retrieval do indeed function simultaneously. We found both significant stimulus-response and significant response-response binding effects, indicating that integration of responses must have occurred at the same time as response retrieval due to feature repetition and vice versa.

## Introduction

Human action control seems to function effortlessly, but research in past decades shows that various more or less complex processes contribute to any action at a given moment in time (Dignath et al., 2019; Frings et al., 2015; Henson et al., 2014; Hommel, 1998; Kiesel et al., 2010; Kunde, 2001; Mayr & Buchner, 2006; Tenpenny, 1995). Based on the ideomotor principle and earlier binding theories (Hommel et al., 2001; Logan, 1988; Schmidt et al., 2016; Shin et al., 2010), the Binding and Retrieval in Action Control framework explicitly separates two core processes that contribute to many classic effects in the action control literature: feature *integration* and *retrieval* due to feature repetition (Frings et al., 2020). If a person responds to a stimulus, stimulus features and response features are integrated in one episodic memory trace that has been termed the *event file* (Hommel, 2004). The next encounter of any of these features then retrieves the event file, affecting further action. If, for example, a stimulus repeats, this triggers retrieval of the integrated response. If the retrieved response matches the currently required response, retrieval facilitates responding; if a different response is required at stimulus repetition, responding is impaired. Such a result pattern indicates binding effects; in this example between stimulus and response.

The characteristics of feature integration and retrieval are typically analyzed in paradigms that rely on both of them working in sequence (e.g., distractor-response binding, Frings et al., 2007; S1R1-S2R2, Hommel, 1998; negative priming, Mayr & Buchner, 2006; Rothermund et al., 2005; response-response binding, Moeller & Frings, 2019a; but see Stoet & Hommel, 1999, for binding effects with a somewhat different logic; see also Dilcher et al., 2021). Hence, in virtually all past studies, the processes contributing to the binding effects can strictly be separated in time. Participants execute a sequence of two responses n-1 and n. Specific features and specific responses are orthogonally varied from n-1 to n, so that measured binding effects can be attributed to feature integration that occurs at the time of response n-1, and retrieval due to feature repetition, affecting response n performance. This separation in past studies can lead to the impression of mutually exclusive processes: past results would not differ if ongoing retrieval prevented simultaneous new integration of the retrieving feature.

Note, however, that an important theoretical assumption regarding binding processes in action control is that integration and retrieval operate simultaneously at every single response (Frings et al., 2020; Hommel et al., 2001): responses affected by retrieval processes are obviously responses that should lead to feature integration. That is, theoretically integration and retrieval processes are not limited to sequential paradigms, only their measurement requires such sequences. Together, there is both a wide consensus that integration and retrieval mechanisms work constantly in parallel and also to date there is no direct evidence for this consensus. Moreover, even with simultaneous integration and retrieval in general, it is still possible that a single feature cannot simultaneously be integrated with one feature while retrieving another one. In the present study we used identical responses that were simultaneously part of stimulus-response binding and response-response binding effects to provide evidence that the same feature can indeed be integrated at the same time it triggers retrieval.

Slightly different paradigms have been used to analyze stimulus-response and response-response binding effects. To measure stimulus-response binding, Moeller et al. (2016) asked participants to respond to individually presented target letters that were surrounded by response-irrelevant geometric shapes. In a prime probe design that required responding to each prime and each probe display, the required response and the identity of the geometric shape orthogonally repeated or changed between prime and probe. Assuming that response and shape were integrated during the prime, repetition of the same shape in the probe led to retrieval of the prime response.

The response-response binding paradigm uses the same logic but includes two independently planned and executed responses in each prime and each probe (Moeller & Frings, 2019a). Participants respond to two individually presented stimuli in each prime and each probe, with the second stimulus only appearing after execution of the first response. Here the assumption is that the two responses are integrated during the prime, so that repeating either of them as the first response in the probe retrieves the other (Moeller & Frings, 2019b).

**Fig. 1:**
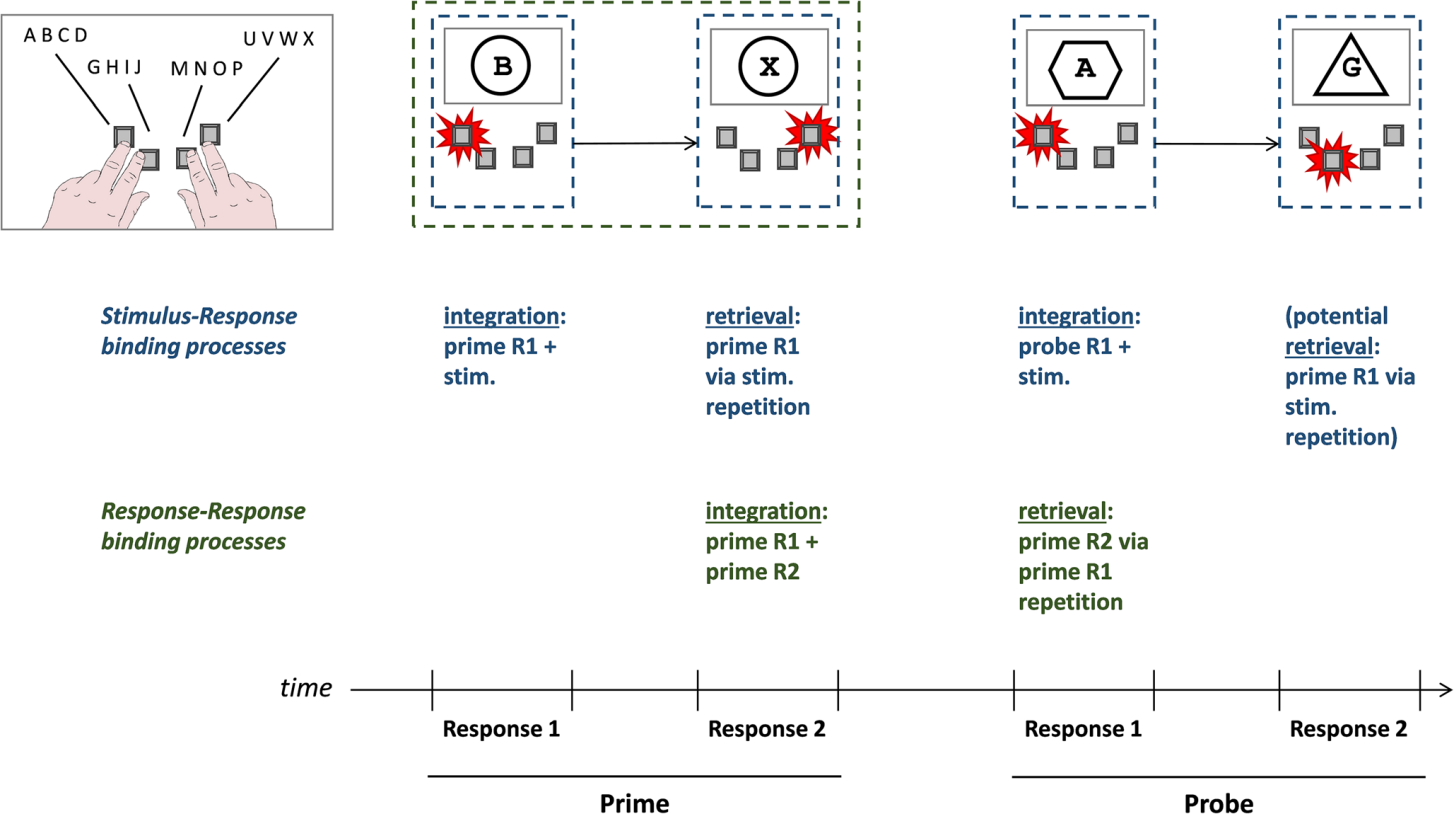
Integration and retrieval processes, involved in stimulus-response and response-response binding effects in a single trial in the experiment. To obtain significant stimulus-response and response-response binding effects, the depicted integration and retrieval processes have to function simultaneously. Participants categorized the central letter by pressing one of four response keys and ignored surrounding geometric shapes. Stimuli were never repeated from prime to probe sequences. Hence, stimulus-response integration that occurred at prime Response 2 was never measured. This is an example of a R1 repetition, R2 change trial in which stimulus-response binding was measured during the prime. This example shows a response change (from prime R1 to prime R2) and stimulus repetition condition

In the present study we assumed that every individual response is integrated with simultaneously occurring stimuli (stimulus-response binding) and also with the directly preceding response (response-response binding; see Fig. 1). In line with hierarchical views on action control, we assume that one event file is formed at each individual response, which can become part of a higher order event file including both prime (or both probe) responses (Moeller & Frings, 2021).^1^ In a sequence of two responses, stimulus-response binding already occurs at the time of the first response (Response 1), while response-response binding can only occur upon execution of the second response (Response 2). Notably, at this point in time (Response 2) stimulus repetition would also lead to retrieval of Response 1. Focusing on the next response pair, repetition of Response 1 from before would here lead to retrieval of Response 2 of the previous pair. At the same time, a new integration of the current stimulus with the executed Response 1 of the current response pair has to be assumed (which could then influence the following Response 2). Hence, if we observe stimulus-response and response-response binding effects in the same trials, this implies that integration and retrieval processes affected behavior simultaneously, with an identical response starting retrieval of an integrated response while being integrated with other features. To anticipate the results, stimulus-response and response-response binding effects were indeed measured simultaneously.

## Experiment

We implemented a response-response binding paradigm and introduced a few changes to also measure stimulus-response bindings either in the prime or in the probe sequence of the response-response binding paradigm. The standard response-response binding paradigm includes two responses (Response 1 and Response 2) during the prime and two responses (Response 1 and Response 2) during the probe of each trial. We then assume that Response 1 and Response 2 identities are integrated during the prime and that repetition of Response 1 identity as probe Response 1 retrieves the other response from the prime, influencing probe Response 2 performance. Statistically, a response-response binding effect is indicated by an interaction of Response 1 relation (repetition vs. change from prime to probe) and Response 2 relation (repetition vs. change from prime to probe).

In the adjusted paradigm, stimulus-response binding was measured during the prime sequence (of Response 1 and Response 2; half of the trials) or during the probe sequence (the other half of the trials). Responses were indicated by individually presented letters and each letter was surrounded by a geometric shape that served as an additional response-irrelevant stimulus. Binding effects resulting from integration and retrieval of this additional stimulus and the response are interpreted as stimulus-response binding effects. Repetition of the additional stimulus is assumed to trigger retrieval of the response that accompanied the stimulus at its last presentation. Hence, stimulus repetition would lead to response facilitation if the response is also repeated but to impairment if different responses are required as Response 1 and Response 2. A significant interaction of response relation (repetition vs. change) and stimulus relation (repetition vs. change) indicates stimulus-response binding.

### Method

#### Participants

Effect sizes (computed as t/sqrt(n)) of response-response binding effects were larger than 0.6 in previous experiments (Moeller & Frings, 2019a: *d* = 0.63 and *d* = 0.84; Moeller & Frings, 2019b: *d* = 0.83). We therefore aimed to find an effect of at least *d* = .6, assuming alpha = .05 (two-tailed) and a power of 1-ß = .90. A power analysis with the program G*Power revealed that 32 participants were required (Faul et al., 2007). This sample size ensured a power of 1−β = .98 for detecting stimulus-response binding effects (assuming *d* = 0.74 as reported for a comparable paradigm of Exp. 1 in Moeller et al., 2016, and a two-tailed test). Thirty-two students (27 female) from the University of Trier took part in the experiment. The median age of the sample was 20 years (range 17–52). All participants reported normal or corrected-to-normal vision and received monetary compensation or partial course credit.

#### Design

For the response-response binding analyses, the design comprised three within-subjects factors: Response R1 Relation (response repetition vs. response change from prime to probe), Response R2 Relation (response repetition vs. response change from prime to probe), and Time of stimulus-response Binding Measurement (during the prime vs. during the probe sequence). For the stimulus-response binding analyses, the design comprised three within-subjects factors: Response Relation (repetition vs. change from R1 to R2), Stimulus Relation (repetition vs. change from R1 to R2), and Time of Measurement (during prime responses vs. during probe responses).

#### Materials

The experiment was conducted using E-prime 2.0. Instructions and stimuli were shown in white on a black background on a standard TFT screen. Target-stimuli were the letters A, B, C, D, G, H, I, J, M, N, O, P, U, V, W, and X. All letters subtended a horizontal visual angle of 0.3°–0.5°, and vertical visual angle of 0.5°. Task-irrelevant stimuli were the outlines of four geometric shapes (square, hexagon, circle, and triangle). These subtended a horizontal visual angle of 2.9°–3.2°, and a vertical visual angle of 2.7°–3.1°. Viewing distance was approximately 60 cm. Participants responded by pressing one of four keys on the computer keyboard.

#### Procedure

The procedure was adapted from Moeller and Frings (2019b) and Moeller et al. (2016). Participants were tested individually in soundproof chambers and instructions were given on the screen. Participants placed middle and index fingers of both hands on the keys S, C, M, and L of a standard computer keyboard, which were marked with the respective letters. Their task was always to press the key corresponding to individually presented letters. For A, B, C, and D, participants responded with their left middle finger, for G, H, I, and J with their left index finger, for M, N, O, and P with their right index finger, and for U, V, W, and X with their right middle finger. All target letters appeared centered on the screen and were surrounded by the outlines of one of the four possible task-irrelevant geometric shapes. Each trial started with the presentation of an asterisk, which after 500 ms was exchanged for a plus sign that lasted for another 500 ms. Then the first prime letter and shape appeared until prime R1 response execution, followed by the second prime letter and shape until prime R2 response execution. A fixation mark appeared for 500 ms and was followed by the first probe letter and shape until the probe R1 response execution. Then the second probe letter and shape appeared until probe R2 response execution. Finally, an asterisk, presented in the middle of the screen, indicated that the next trial had started. In the case of an incorrect response R1 or R2 in prime or probe, an error message appeared for 1,500 ms immediately following the erroneous response, reminding the participant to respond as quickly as possible but without errors. After every 40 trials participants were allowed to take a short break, after which they resumed the task in their own time.

Response R1 relation from prime to probe (repetition vs. change) was varied orthogonally to response R2 relation from prime to probe (repetition vs. change), while target letters did not repeat within a single trial. In R1 repetition trials, the same response was required to the letter indicating prime response R1 and the one indicating probe response R1. In R1 change trials, different responses were required to the stimulus indicating prime response R1 and the one indicating probe response R1. In R2 repetition trials, the same response was required to the stimulus indicating probe response R2 and the one indicating prime response R2. In R2 change trials, different responses were required to the stimulus indicating probe response R2 and the one indicating prime response R2. This resulted in the four different conditions R1repetition/R2repetition, R1repetition/R2change, R1change/R2repetition, and R1change/R2change, which are necessary to measure response-response binding effects. Orthogonally to these the factors response relation from R1 to R2 and stimulus relation from R1 to R2 were varied for the measurement of stimulus-response binding effects. In response repetition trials, the same response was required as R1 and R2 (in the prime or in the probe, respectively). In response change trials, different responses were required as R1 and R2. In stimulus repetition trials, the same shape surrounded the letter indicating R1 and the one indicating R2, and in stimulus change trials different shapes surrounded the letters indicating R1 and R2. That is, four different conditions were used to measure stimulus-response bindings: response repetition/stimulus repetition, response repetition/stimulus change, response change/stimulus repetition, and response change/stimulus change. In half of the trials, stimulus-response binding was measured during the prime and in the other half of the trials stimulus-response binding was measured during the probe. In the primes and probes that were not used to measure stimulus-response binding, task-irrelevant shapes always changed between R1 and R2, and also differed from those shapes presented in the respective probe (or prime) of the trial. For examples of all conditions, see Table 1. Stimulus or response repetition was also possible from probe R2 of trial n-1 to prime R1 of trial n, likely affecting individual prime R1 performance. However, since these repetitions were not systematically varied, any integration and retrieval only increased the variance of R1 performance, and binding effects could not be measured. The experimental block consisted of 256 experimental trials. Before the experimental block started, participants practiced their task for 32 trials (subsample of the experimental trials).

**Table 1:**
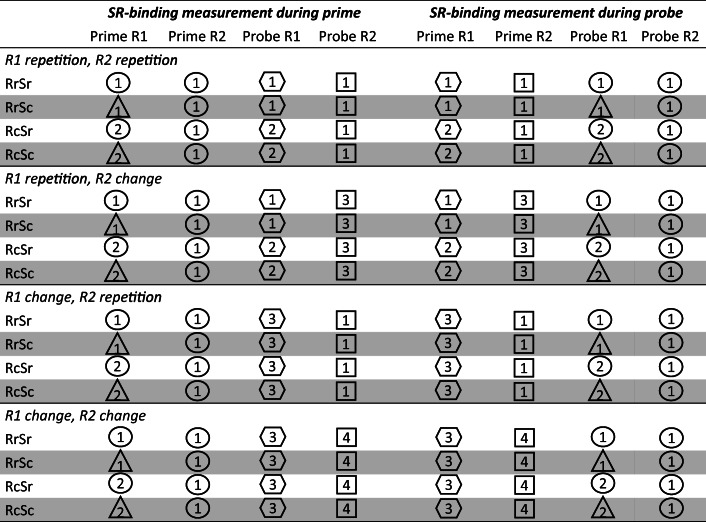
Examples for stimulus presentations in prime R1, prime R2, probe R1, and probe R2 as a function of response-response binding and stimulus-response(SR) binding conditions in the experiment. For the sake of clarity, numbers represent required responses

Note that target letters (which indicate the required responses) actually never repeated within a single trial

*RrSr* response repetition/stimulus repetition, *RrSc* response repetition/stimulus change, *RcSr* response change/stimulus repetition, *RcSc* response change/stimulus change

### Results

#### Response-response binding

The dependent variable of interest for the response-response binding effect was performance in probe R2. For the response time (RT) analysis, we considered only those trials with correct responses in both the prime and the probe. The rate for at least one error in the prime responses was 5.3%. Probe error rates were 2.5% for R1 and 2.4% for R2. RTs that were more than 1.5 interquartile ranges above the third quartile of the RT distribution of the participant (Tukey, 1977) and RTs that were shorter than 200 ms were excluded from the RT analysis. Due to these constraints, 14.4% were excluded. For mean RTs and error rates (ERs), see Table 2.

**Table 2:** Mean response times (in ms) and mean error rates (in percent) for probe responses R2, as a function of R1 relation from prime to probe, R2 relation, and time of stimulus-response binding measurement (prime vs. probe)

	Stimulus-response binding measurement in			
	prime		Probe	
	R2 repetition	R2 change	R2 repetition	R2 change
*Probe R2*				
R1 change	833 (3.4)	843 (1.8)	787 (1.6)	800 (1.8)
R1 repetition	744 (1.2)	860 (3.0)	742 (2.2)	804 (4.0)
Priming Effect	89 (2.2)	-17 (-1.2)	45 (-0.6)	-4 (-2.2)

**Fig. 2:**
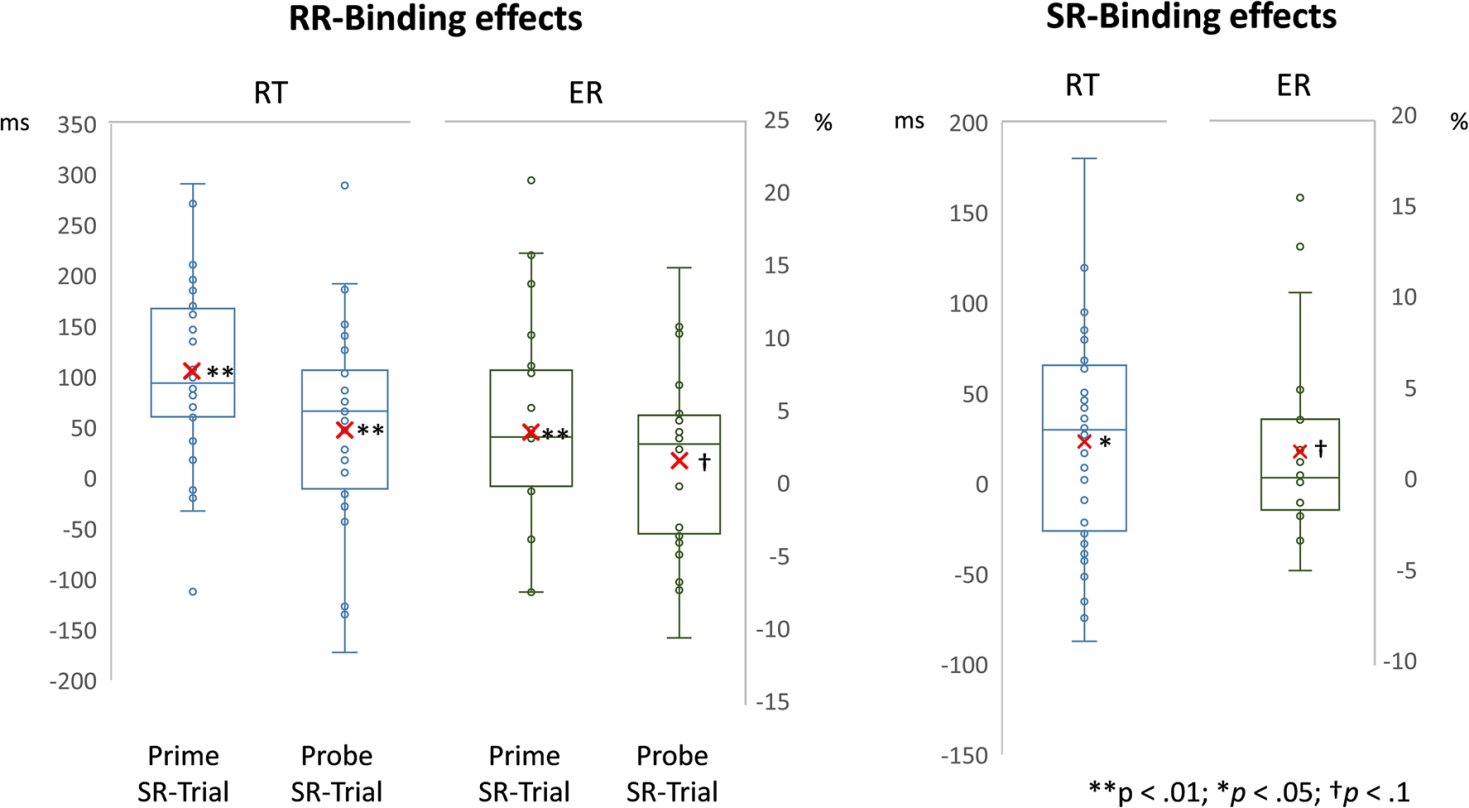
Distribution of the individual response-response(RR) binding effects (left-hand side) and stimulus-response(SR) binding effects (right-hand side) for reaction times (RTs) in ms and error rates (ERs) in %. Mean binding effects are marked with a red x. Response-response binding effects were calculated as R1 change minus R1 repetition RTs of probe R2 for R2 change trials, subtracted from R1 change minus R1 repetition RTs of probe R2 for R2 repetition trials ([R1change/R2repetition − R1repetition/R2repetition] − [R1change/R2change − R1repetition/R2change]). SR binding effects were calculated as the difference between stimulus repetition effects in response repetition and response change trials ([response repetition/stimulus change − response repetition/stimulus repetition] − [response change/stimulus change − response change/stimulus repetition])

In a 2 (R1 relation from prime to probe: repetition vs. change) × 2 (R2 relation from prime to probe: repetition vs. change) × 2 (time of stimulus-response binding measurement: prime vs. probe) MANOVA on probe response R2 RTs with Pillai’s trace as the criterion, all main effects were significant; time of stimulus-response binding measurement: *F*(1,31) = 15.76, *p* < .001, *η*_p_^2^ = .34, R1 relation: *F*(1,31) = 11.71, *p* = .002, *η*_p_^2^ = .27, R2 relation: *F*(1,31) = 68.85, *p* < .001, *η*_p_^2^ = .69. Importantly, the interaction of R1 and R2 relation was also significant, *F*(1,31) = 37.31, *p* < .001, *η*_p_^2^ = .55, indicating binding between responses. This binding effect was further modulated by the time of stimulus-response binding measurement, *F*(1,31) = 7.85, *p* = .009, *η*_p_^2^ = .20, indicating a larger response-response binding effect if stimulus-response binding was measured during the prime. We return to this difference in the *Discussion*. Notably, individual analyses indicated significant response-response binding effects both for measurement during the prime, *F*(1,31) = 46.96, *p* < .001, *η*_p_^2^ = .60, and measurement during the probe, *F*(1,31) = 8.38, *p* = .007, *η*_p_^2^ = .21 (Fig. 2, left-hand side). The interaction of R2 relation and time of stimulus-response binding measurement, *F*(1,31) = 5.68, *p* = .023, *η*_p_^2^ = .16, was also significant.

In the same analysis on ERs, the main effect of R2 relation came close to significance, *F*(1,31) = 2.99, *p* = .094, *η*_p_^2^ = .09. The interaction of R1 relation and R2 relation was significant, *F*(1,31) = 11.71, *p* = .002, *η*_p_^2^ = .27, again indicating binding between the responses. The interaction of R1 relation and time of stimulus-response binding measurement was also significant, *F*(1,31) = 9.34, *p* = .005, *η*_p_^2^ = .23. None of the other effects were significant, *F*s < 2.2, *p*s > .15, *η*_p_^2^s < .07.

#### Stimulus-response binding

The dependent variable of interest for the stimulus-response binding effect was response R2 performance either in the prime or in the probe (depending on the time of stimulus-response binding measurement). For the RT analysis, we considered only those trials with correct responses to R1 and R2 in this prime or probe, respectively. The rate for R1 errors was 2.6% in stimulus-response binding prime trials and 2.5% in stimulus-response binding probe trials. R2 error rates were 3.0% in stimulus-response binding prime trials, and 2.4% in stimulus-response binding probe trials. RTs that were more than 1.5 interquartile ranges above the third quartile of the RT distribution of the participant (Tukey, 1977) and RTs that were shorter than 200 ms were excluded from the RT analysis. Due to these constraints, 9.9% of the trials were excluded. For mean RTs and ERs, see Table 3.

**Table 3:** Mean response times (in ms) and mean error rates (in percent) for R2 responses (in the prime or probe respectively), as a function of response relation from R1 to R2, and stimulus relation

	Response repetition	Response change
Prime/Probe R2		
Stimulus change	736 (2.7)	868 (2.7)
Stimulus repetition	708 (2.0)	864 (3.4)
Priming Effect	28 (0.7)	4 (-0.7)

In a 2 (response relation from R1 to R2: repetition vs. change) × 2 (stimulus relation from R1 to R2: repetition vs. change) × 2 (time of stimulus-response binding measurement: prime vs. probe) MANOVA on prime/probe response R2 RTs with Pillai’s trace as the criterion, all main effects were significant, *F*(1,31) = 7.91, *p* = .008, *η*_p_^2^ = .20, for time of stimulus-response binding measurement, *F*(1,31) = 105.78, *p* < .001, *η*_p_^2^ = .77, for response relation, and *F*(1,31) = 4.30, *p* = .046, *η*_p_^2^ = .12, for stimulus relation. Importantly, the interaction of response relation and stimulus relation was also significant, *F*(1,31) = 5.18, *p* = .030, *η*_p_^2^ = .14, indicating stimulus-response binding (Fig. 2, right-hand side). This binding effect was not modulated by the time of stimulus-response binding measurement, *F*(1,31) < 1, *p* = .553, *η*_p_^2^ = .01. Follow-up analyses confirmed the stimulus-response binding pattern: stimulus repetition led to faster response times only if the response was repeated, *t*(31) = 3.19, *p* = .003, *d* = 0.56, but not if the response changed from R1 to R2, *t*(31) = 0.46, *p* = .652, *d* = 0.08. The interaction of stimulus relation and time of stimulus-response binding measurement, *F*(1,31) = 4.66, *p* = .039, *η*_p_^2^ = .13, was also significant.

In the same analysis on ERs, only the interaction of response relation and stimulus relation approached significance, *F*(1,31) = 3.65, *p* = .065, *η*_p_^2^ = .11, with a pattern suggesting stimulus-response binding. None of the other effects were significant, *F*s < 3, *p*s > .1, *η*_p_^2^s < .09.

## Discussion

We analyzed stimulus-response and response-response binding effects in the same trials and found evidence for both binding effects. It is important that this was the case, even though individual responses needed at the same time to be integrated with a stimulus and retrieve a different response, and vice versa. This is evidence for binding effects that relied on simultaneous integration and retrieval processes.

Note that we found a larger response-response binding effect if stimulus-response binding was measured during the prime than if it was measured during the probe. This difference is likely due to response-response binding being boosted by response repetitions during the probe responses if stimulus-response binding was measured during the prime but not if stimulus-response binding was measured during the probe (see Table 1). If stimulus-response binding was measured during the probe, half of the trials in each of the response-response binding conditions (R1repetition/R2repetition, R1repetition/R2change, R1change/R2repetition, R1change/R2change) included response repetition between probe responses R1 and R2. That is, if stimulus-response binding was measured during the probe, any effect of response repetition between probe responses R1 and R2 led to generally faster responding in all conditions contributing to the response-response binding effect. By contrast, if stimulus-response binding was measured during the prime, response repetitions between probe responses R1 and R2 occurred only in the response-response binding condition that repeated both R1 and R2 from prime to probe. Here, half the trials included response repetitions between probe responses, leading to faster mean RTs compared to the other three conditions, in turn boosting the response-response binding effect. Therefore, we mainly rely on the response-response binding effect in the trials measuring stimulus-response binding during the probe and are cautious with an interpretation of the response-response binding effect in the rest of the trials. Most importantly, the response-response binding effect was also significant if it was unaffected by response repetition effects.

Looking at a more detailed timeline, retrieval and integration likely occurred sequentially in the prime sequence: stimulus repetition in prime R2-displays could retrieve a response that was then (upon execution) integrated with the previous response. Yet in probes, a sequence is less clear. Integration of the stimulus in the probe R1-display with the executed response is assumed to occur at response execution (Hommel, 2005). This same response execution could also trigger retrieval of a response (that was executed in the prime). That is, even if we expect sequential retrieval and binding in the present prime responses, simultaneous binding and retrieval must have occurred during the first probe response.

Hence, in line with theoretical assumptions (Frings et al., 2020; Hommel et al., 2001), integration and retrieval processes do not only affect behavior if they function sequentially, with binding at a first response, enabling its corresponding retrieval mechanism to start at the second response. In fact, a feature that is being integrated with other features can at the same time trigger retrieval of previously integrated features. This demonstrates that binding and retrieval processes affect action not only in sequential paradigms, including two responses, but virtually in all action control paradigms and other situations in which humans act. We have to remind ourselves (and other researchers) that we separate binding and retrieval in the laboratory so as to measure these processes independently (e.g., Frings & Rothermund, 2011; Moeller & Frings, 2019c), but that this does not mean that the results these paradigms yield can only be applied to sequential responses.

We assume that actions were hierarchically coded in the present paradigm, each response being integrated in one event file and prime (probe) response pairs being integrated in higher-order events. Even though this might be the closest to the originally assumed event files (Hommel et al., 2001), it is only one possibility to account for overlapping integration and retrieval in the present study. Another possibility is that all stimuli and responses in a trial are integrated in the same non-hierarchically structured event file. Under this conception, the present results would indicate that event files continuously build up over time and that they spread *across* feature integration and retrieval. This would imply that not an event is integrated and can be retrieved later on, but rather features are integrated and can retrieve each other within the course of one event. We prefer the former interpretation because it is in line with a large body of action control and event representation literature (e.g., Vallacher & Wegner, 1987; Yamaguchi & Logan, 2014; Zacks & Swallow, 2007), while the event concept seems to lose its meaning with the latter.

With integration and retrieval affecting behavior simultaneously, we also have to consider mutual influences between the mechanisms. For example, it may not only be the present feature that is integrated in the current event file, but retrieved features might be integrated as well. This resembles what has been suggested for episodic encoding and retrieval in memory (Tulving et al., 1994), underlining structural similarities between event coding and memory paradigms. Yet, encoding and retrieval in memory (e.g., Allan & Allen, 2005; Naveh-Benjamin et al., 1998; Naveh-Benjamin et al., 2006) and binding and retrieval in action control (e.g., Hommel, 2005; Hommel et al., 2014; Moeller & Frings, 2014) react very differently to potential modulators. Memory encoding and action retrieval are susceptible to modulations, while memory retrieval and stimulus-response integration are decisively less affected. Specific research will be necessary to better understand the relation between memory and event-coding effects.

To conclude, here we present evidence that two core processes in human action control, namely binding and retrieval, affect behavior simultaneously although they are separated in sequential paradigms for measurement reasons. This is in line with theoretical assumptions in stimulus-response binding, and underlines that binding and retrieval processes affect everyday actions that typically do not occur in sequential prime-probe designs.

## Data Availability

The data for the experiment reported here will be made available on PsychArchives after publication; none of the experiments was preregistered.

## Footnotes

1 Note that our focus on individual responses and response pairs as events only includes a small part of a larger hierarchy.
